# Intra-specific variation in genome size in maize: cytological and phenotypic correlates

**DOI:** 10.1093/aobpla/plv138

**Published:** 2015-12-07

**Authors:** María Florencia Realini, Lidia Poggio, Julián Cámara-Hernández, Graciela Esther González

**Affiliations:** 1 Instituto de Ecología, Genética y Evolución (IEGEBA)-Consejo Nacional de Investigaciones Científicas y Técnicas (CONICET) and Laboratorio de Citogenética y Evolución (LaCyE), Departamento de Ecología, Genética y Evolución, Facultad de Ciencias Exactas y Naturales, Universidad de Buenos Aires, Ciudad Autónoma de Buenos Aires, Argentina; 2 Cátedra de Botánica Agrícola, Facultad de Agronomía, Universidad de Buenos Aires, Ciudad Autónoma de Buenos Aires, Argentina

**Keywords:** DAPI staining, DNA content variation, FISH, Guarani's maize landraces, heterochromatic knobs, karyotype parameters

## Abstract

Genome size variation accompanies the diversification and evolution of many plant species. Relationships between DNA amount and phenotypic and cytological characteristics form the basis of most hypotheses that ascribe a biological role to genome size. The goal of the present research was to investigate the intra-specific variation in the DNA content in maize populations from Northeastern Argentina and further explore the relationship between genome size and the phenotypic traits seed weight and length of the vegetative cycle. Moreover, cytological parameters such as the percentage of heterochromatin as well as the number, position and sequence composition of knobs were analysed and their relationships with 2C DNA values were explored. The populations analysed presented significant differences in 2C DNA amount, from 4.62 to 6.29 pg, representing 36.15 % of the inter-populational variation. Moreover, intra-populational genome size variation was found, varying from 1.08 to 1.63-fold. The variation in the percentage of knob heterochromatin as well as in the number, chromosome position and sequence composition of the knobs was detected among and within the populations. Although a positive relationship between genome size and the percentage of heterochromatin was observed, a significant correlation was not found. This confirms that other non-coding repetitive DNA sequences are contributing to the genome size variation. A positive relationship between DNA amount and the seed weight has been reported in a large number of species, this relationship was not found in the populations studied here. The length of the vegetative cycle showed a positive correlation with the percentage of heterochromatin. This result allowed attributing an adaptive effect to heterochromatin since the length of this cycle would be optimized via selection for an appropriate percentage of heterochromatin.

## Abstract (precis)

In the maize landraces from Northeastern Argentina inter and even intra-populational genome size variations were detected. Moreover, high variation in number, positions, percentage of heterochromatin as well in size and sequence compositions of knobs were detected. Since knobs would be an important cause of the observed differences in DNA content, the absence of a significant relationship between the percentage of heterochromatin and genome size suggests that other non-coding repetitive DNA sequences contribute to genome size variation. The positive correlations between the length of the vegetative cycle and percentage of heterochromatin found allowed us to attribute an adaptive effect to the heterochromatin, since the vegetative cycle time would be optimized via selection for an appropriate percentage of heterochromatin.

## Introduction

It is well known that genome size varies among species and their diversification accompanies the evolution of many groups of plants (Bennett and Leitch 2005; Leitch *et al*. 2005; Gregory *et al*. 2007; Leitch and Leitch 2013; Poggio *et al*. 2014). The increase of genome size arises predominantly through polyploidy and amplification of non-coding repetitive DNA, heterochromatin and retrotransposons. These mechanisms are counterbalanced by mechanisms of decrease in genome size, genome downsizing, and have been shown to involve recombination-based processes (Soltis *et al*. 2003; Wicker *et al*. 2003; Bennetzen *et al*. 2005; Grover and Wendel 2010). Several studies demonstrated that there exists intra-specific and even intra-populational genome size variations (revisited in Greilhuber and Leitch 2013). *Zea* is an interesting model because it shows both inter- and intra-specific variations in DNA amount (Poggio *et al*. 1998; Rosato *et al*. 1998; SanMiguel and Bennetzen 1998; Bennett and Leitch 2005; Meagher and Vassiliadis 2005; Díez *et al*. 2013). It was proposed that this variation is principally due to differences in the heterochromatin amount, mainly located in chromosome blocks, named knobs, as well as the presence of B-chromosomes (Laurie and Bennett 1985; Rayburn *et al*. 1985; Tito *et al*. 1991; Poggio *et al*. 1998; Rosato *et al*. 1998; González and Poggio 2011). The variation in the DNA content has also been attributed to differences in the interspersed DNA amount, such as the retrotransposon families, which in maize make up over 70 % of the nuclear genome (SanMiguel and Bennetzen 1998; Meyers *et al*. 2001). In *Zea,* genome size is highly related to the inter- and intra-specific variations in the number and size of heterochromatic knobs (revisited in Poggio *et al*. 1998). These knobs occur in all *Zea* species with 2*n* = 20 and have been observed in 34 distinct cytological locations, varying in size and number across maize races and their wild relatives (Kato 1976; McClintock *et al*. 1981; Tito *et al*. 1991; Rosato *et al*. 1998; González and Poggio 2011; González *et al*. 2013). They are composed primarily of two tandem-repeated sequences, the 180-bp and TR-1, varying between a few thousand to millions of repeats (Peacock *et al*. 1981; Ananiev *et al*. 1998). 4′,6-Diamidino-2-phenylindole (DAPI) staining and fluorescent *in situ* hybridization (FISH) allowed us to reveal the chromosome location and the sequence composition of the knobs (Albert *et al*. 2010; González and Poggio 2011; Mondin *et al*. 2014).

Several studies reported positive relationships between the DNA amount and phenotypic characteristics such as seed mass and plant flowering time (Bennett 1987; Grime and Mowforth 1982; Ohri and Pistrick 2001; Knight *et al*. 2005; Beaulieu *et al*. 2007; Greilhuber *et al*. 2007; Greilhuber and Leitch 2013). These correlations form the basis of most hypotheses that ascribe a biological role to genome size. It was Bennett who coined the term ‘nucleotype’ to describe ‘that condition of the nucleus that affects the phenotype independently of the informational content of the DNA’ (Bennett 1971, 1972). The correlation between genome size and ecological variables in maize and its wild relatives has been examined (Laurie and Bennett 1985; Rayburn *et al*. 1985; Rayburn and Auger 1990*a*, *b*; Díez *et al*. 2013). A negative correlation between genome size and altitude/latitude has been reported, suggesting that genome size could be related to the rapid growth and early flowering in the shorter growing seasons, typical of cool regions (Laurie and Bennett 1985; Rayburn 1990; Rayburn and Auger 1990*a*, *b*; Rayburn *et al*. 1994; Rosato *et al*. 1998: Poggio *et al*. 1998; Bennett and Leitch 2005).

In northern Argentina, more than 60 morphological native maize races have been described, and up to 15 of these can be found in indigenous settings from the subtropical forests of the Misiones Province (Melchiorre *et al*. 2006; Cámara Hernández *et al*. 2011). In this restricted geographic area, Guarani's indigenous communities cultivate maize landraces with little or no input from commercial germplasm (Bracco *et al*. 2012). It is interesting to point out that these Guarani's landraces show remarkable phenotypic differences and high genetic diversity (Melchiorre *et al*. 2006; Cámara Hernández *et al*. 2011; Bracco *et al*. 2012).

In the present work in maize Guarani's landraces, the variation in 2C values was measured by flow cytometry and knob heterochromatin was evaluated using DAPI banding and FISH. The goal of the present research was to investigate intra-specific variation in the DNA content and further explore the relationship between genome size and cytological parameters such as the percentage of heterochromatin, number, position and sequence composition of knobs, as well as the relationships with phenotypic traits such as seed weight and the length of the vegetative cycle. The inter-populational genome size variations and their correlation with cytogenetic and phenotypic traits could shed light on the biological importance of such variation and their potential adaptive significance.

## Methods

### Plant materials

Twenty populations of maize all belonging to Guarani's maize landraces described from Northeastern Argentina (NEA) were collected from original indigenous populations in Misiones Province, Argentina (Table 1). The specimens were deposited at the seed bank of the Vavilov Laboratory (Facultad de Agronomía, Universidad de Buenos Aires).

**Table 1.** Local names, VAV and collection sites and altitude of cultivation. Ref.^1^ Corneal grains (popcorn); ^2^ floury grains (floury); ^3^ floury grains with corneal periphery.

Maize landraces (local names)	VAV	Collection sites	Altitude (m a.s.l.)
Rosado (Avatí Yuí)	6565^2^	Colonia Její, Depto. Guaraní.	329
Variegado (Avatí Tové)	6557^2^	Paraje Paraíso, Depto. San Pedro.	525
Overo (Avatí Pará)	6559^2^	Paraje Paraíso, Depto. San Pedro.	525
Tupí Amarillo (Avatí Tupí)	6563^3^	Aldea Pindó Poty, Depto. Guarani.	329
Tupí Blanco (Avatí Tupí)	6592^3^	Pozo Azul, Depto. San Pedro.	211
Tupí Blanco (Avatí Tupí)	6851^3^	Aldea Kaqvy Mitaú Rupá, Ruiz de Montoya, Depto. Libertador General San Martín.	240
Pororó Chico (Avatí Pororó)	6575^1^	Aldea MBY’A Guaraní, Guavyra Pory, Paraje Paraíso, Depto. San Pedro.	525
Pipoca Colorado	6607^1^	Aldea Alecrín, Depto. Eldorado.	211
Pipoca Colorado	6567^1^	Aldea Pozo Azul, Depto. Eldorado.	211
Colorado (Avatí Pytá í)	6837^3^	Aldea Perutí, El alcázar, Depto. Libertador General San Martín.	591
Colorado (Avatí Pytá í)	6573^3^	Comunidad Miri, Aldea MBÝA Guaraní, Depto. Candelaria (Municipio de Sta. Ana).	98
Azul (Avatí Ovy)	6564^2^	Colonia Její, Depto. Guaraní.	329
Pipoca Amarillo	6568^1^	Aldea Pozo Azul, Depto. Eldorado.	211
AmarilloAngosto (Avatí Mitá í)	6556^2^	Aldea MBYA Guaraní, Guavyra Pory, Paraje Paraíso, Depto. Eldorado.	535
Amarillo Ancho (Avatí Yu)	6569^2^	Pozo azul, Depto. Eldorado.	211
Blanco Angosto (Avatí Pará í)	6574^2^	Comunidad Miri, Aldea MBÝA Depto. Guaraní.	98
Blanco Ancho (Avatí Morotí)	6560^2^	Aldea Chiripa Guaraní, Pindó Poty, Depto. Guaraní.	329
Pororó Grande (Avatí Pororó)	6827^1^	Aldea Guavyra Pory, Paraje Paraíso, Depto. San Pedro.	535
Pororó Grande (Avatí Pororó)	6826^1^	Aldea Guavyra Pory, Paraje Paraíso, Depto. San Pedro.	535
Pororó Grande (Avatí Pororó)	6562^1^	Aldea Chiripa Guaraní, Pindó Poty, Depto. Guaraní.	329

### Methods

Seeds were germinated at 28 °C, for 2–3 days, in Petri dishes containing wet filter paper. Primary root tips, 0.5–1 cm in length, were pre-treated with 8-hydroxiquinoline (0.02 M) for 5 h at room temperature. Then they were fixed in 3 : 1 (ethanol : acetic acid) and stored at 4 °C until use. Seedlings were grown until adult leaves are produced for genome size measurement.

### DNA content measurement

**Figure 1.**
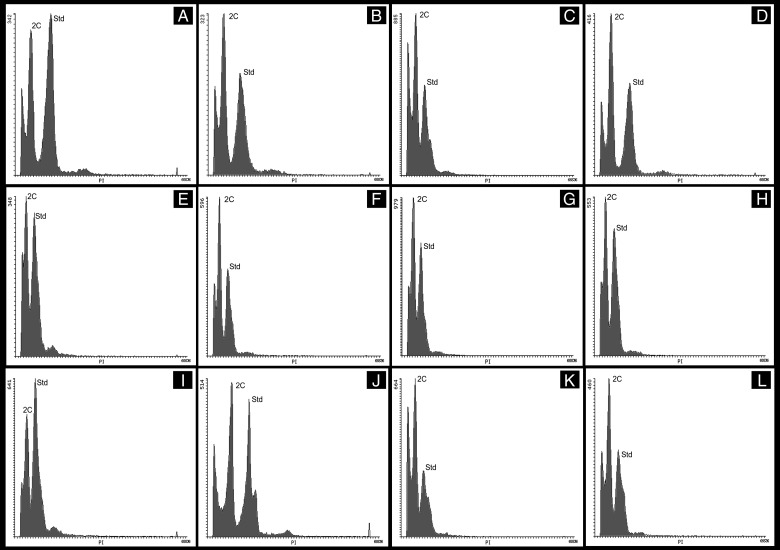
Fluorescence histograms of individuals of six maize populations. Histograms of (A and B) VAV6557, (C and D) VAV6568, (E and F) VAV6556, (G and H) VAV6569, (I and J) VAV6574 and (K and L) VAV6592. 2C indicates G0/G1 peaks from maize samples and Std indicates G0/G1 peaks from the standard *P. sativum*.

The 2C DNA content was measured in three to five individuals of each corncob and two to five corncobs per population, with thee replicates per individual. The cell nucleus were stained with propidium iodide (PI). *Pisum sativum* cv. citrad (9.09 pg), used as internal standard, was kindly provided by Dr J. Doležel from the Institute of Experimental Botany, Sokolovská, Czech Republic (Doležel *et al*. 2007). For each individual, 100 mg of fresh leave samples were co-chopped with 50 mg of *P. sativum* leaves in a Petri dish with 0.5 mL of buffer Otto I (citric acid 0.1 M and 0.5 % v/v of Tween 20), using a stainless-steel razor blade. The sample was filtered through a nylon mesh (45 µm pore size) and then 0.5 mL of buffer Otto II (0.4 M Na_2_HPO_4_ · 12H_2_O) supplemented with PI (50 µg mL^−1^ final concentration) and RNase (50 µg mL^−1^ final concentration) were added. The samples were incubated in the dark for 40 min. Flow cytometry was performed at Instituto Nacional de Tecnología Agropecuaria (INTA-Catelar) with a CyFlow Ploidy Analyzer Cytometer (Partec). We adjusted the gain to 400 and the sample speed to 0.4 µL s^−1^. The samples were run until 5000 nuclei were scored. The DNA content was estimated from gated fluorescence histograms of the PI area (Fig. 1). Data analysis was performed using the software Flowing 2.5.0 (www.flowingsoftware.com). Genome size (2C DNA, in picograms) was determined comparing the peak of the sample to the peak of the standard according to Doležel *et al*. (2007). All samples with a coefficient of variance ≤5 were included in the present study. Conversion from picograms to megabase pairs was done according to Doležel *et al*. (2003).

### Chromosomal preparations

In 12 populations, mitotic metaphase preparations were performed. Fixed root tips were treated with an enzymatic solution (2 % cellulose Onozuka R10 and 20 % Pectinase) for 1 h at 37 °C. Slides with well-spread metaphases were selected by contrast-phase microscopy. After freezing to remove the coverslips, the slides were air-dried and stored at 4 °C until use.

### 4′,6-Diamidino-2-phenylindole staining

This technique was carried out according to Summer (1990). Slides were washed in McIlvaine buffer (citric acid–NaHPO buffer, pH 7), and then stained with 1 µg mL^−1^ DAPI. Slides were incubated in a moist box at 20 °C, in the dark, for 25 min. After staining, the preparations were briefly washed with distilled water, McIlvaine buffer and then distilled water again. Slides were mounted in Mcllvaine buffer and sealed with rubber solution.

### DNA probes

For FISH analysis, the maize knob sequences, 180-bp and TR-1(350 bp), were used as probes. Sequences were obtained from GenBank (http://www.ncbi.nlm.nih.gov/), and were isolated and amplified from total genomic DNA of maize by polymerase chain reaction (PCR) methods. The primers were designed using the Primer3 program (version 0.6) provided by the Whitehead Institute for Biomedical Research & Howard Hughes Medical Institute, USA (http://frodo.wi.mit.edu/primer3/primer3_code.html).

Theses probes were biotin and digoxigenin-labelled, by PCR, using fluorescently conjugated nucleotide 16-biotin-dUTP (Sigma) and 11-digoxigenin-dUTP (Roche).

### Fluorescence *in situ* hybridization

The technique was carried out according to Cuadrado and Jouve (1995), with minor modifications (Poggio *et al*. 1999). Slide preparations were incubated in 100 µg mL^−1^ of RNAse in 2× saline sodium citrate (2 × SSC) for 1 h at 37 °C in a humidified chamber and washed three times in 2 × SSC for 5 min each at room temperature. The slides were post-fixed in freshly prepared 4 % (w/v) paraformaldehyde in distilled water for 10 min and then washed in 2 × SSC for 15 min at room temperature. Then, the preparations were dehydrated in a graded ethanol series and air-dried. The hybridization mixture consisted of 50 % (w/v) deionized formamide, 10 % (w/v) dextran sulfate, 0.1 % (w/v) sodium dodecyl sulfate and 0.3 mg mL^−1^ of salmon sperm in 2 × SSC, then 100 ng of labelled probe was added to 30 µL of hybridization mixture for each slide. The hybridization mixture was denatured for 15 min at 75 °C, loaded onto the slides and covered with a plastic coverslip. The slides were placed on a thermocycler at 75 °C for 7 min, 45 °C for 10 min and 38 °C for 10 min. Then the slides were incubated overnight at 37 °C. Following hybridization, coverslips were carefully floated off by placing the slides in 2 × SSC at 42 °C for 3 min each and then given an astringent wash in 20 % formamide in 0.1 × SSC at 42 °C for 10 min. The slides were washed in 0.1 × SSC at 42 °C for 5 min, 2 × SSC at 42 °C for 5 min, and then transferred to buffer (4 × SSC, 0.2 % (v/v) Tween 20) at 42 °C for 5 min and at room temperature for 1 h in the same buffer. The slides were incubated in the detection buffer containing a solution of 2.5 % bovine serum albumin and the corresponding detection antibody [Cy3 conjugate or anti-digoxigenin-fluorescein isothiocyanate (FITC)] for 1 h at 37 °C and washed three times in 4 × SSC/Tween buffer for 10 min each at room temperature. Slides were counterstained with 1 µg mL^−1^ of DAPI in 4 × SSC/Tween buffer for 40 min at room temperature and then mounted in anti-fade solution (Vector Lab). Slides were examined with a Carl Zeiss Axiophot epifluorescence microscope with appropriate Carl Zeiss filters coupled with a Leica DC 250 digital camera and with an image analyser Leica IM 1000. The location of hybridization signals and DAPI-positive zones were based on the observation of 10 complete metaphases for each analysed individual.

### Karyotypic parameters

The percentage of knob heterochromatin was calculated in 12 populations as the percentage of total chromosome length, using the freeware program MicroMeasure3.3 (http://www.colostate.edu/Depts/Biology/MicroMeasure). Measurements were based on at least 10 cells from each individual and at least 12 individuals of each population. For the elaboration of a representative idiogram of chromosome knob positions, the identification and localization of the knobs were performed in nine populations.

### Phenotypic parameters

The seed mass was calculated as the average weight of 10 kernels of each corncobs of each population. The seeds were weighed in a digital scale, performing three replicates per corncob.

The length of the vegetative cycle was estimated by Ing Pedro Melchiorre as in Melchiorre *et al*. (2006).

### Statistical analysis

The analysis of variation of 2C values among populations was performed by analysis of variance with full nesting using generalized linear mixed models. The variance was modulated with VarExp. Multiple comparisons test was performed by Fisher's least significant difference test (Fisher 1932).

2C DNA intra-populational variation was analysed. The intervals of confidence (ICs) for corncobs and individuals were obtained. The intra-populational variability was considered significant when 0 was not included in the IC. The data of percentage of heterochromatin and the number of knobs were correlated. The means of the percentage of knob heterochromatin and 2C DNA amount were correlated. The means of vegetative cycle and seed mass were correlated with the mean of genome size. The means of percentage of knob heterochromatin and vegetative cycle were also correlated. All correlations were performed using the Spearman coefficient. These statistical analyses were considered significant at *P*-values <0.05, and were performed using the program Infostat, FCA, National University of Córdoba (Di Rienzo *et al*. 2012). For the nested and unbalanced design, the R package (R Development Core Team 2012) was used.

## Results

In the 20 populations of the *Guarani's* maize studied, the DNA amount (2C value) varied from 4.62 to 6.29 pg, representing 36.15 % of inter-populational variation. Significant differences in the 2C DNA content (*P* = <0.0001; *F*_19,__437_ = 8.29) were detected among populations (Table 2). The minimum and maximum values of 2C DNA content (pg, fold) from each population are given in Table 2. A significant intra-populational variability was detected among corncobs (IC: 0.12–0.34) and individuals from the same corncobs (IC: 0.26–0.36).

**Table 2.** Mean (±SD) 2C genome size (in pg) of each maize population, 1C value (in Mbp), range of genome size, percentage of knob heterochromatin, vegetative cycle and seed mass. Populations with different letters have significantly different means of genome size (*P* < 0.05) *Data of vegetative cycle and seed mass were kindly donated by Ing. Agr. Melchiorre from the Facultad de Agronomía, Universidad de Buenos Aires.

Maize landraces/vouchers	2C DNA (pg) mean (±SD)	1C value (Mbp)	Minimum and maximum 2C DNA content (pg)/folds	Percentage of knob heterochromatin	Vegetative cycle (days)*	Seed mass (g)
Tupí Amarillo/VAV6563	6.29 ± 0.59^A^	3076	4.63–7.56 pg/1.63-fold	10.33–16.10 %	82	2.59*
Tupí Blanco/VAV6851	6.07 ± 0.21^AB^	2968	5.79–6.35 pg/1.10-fold		–	2.38
Pororó Grande/VAV6826	6.04 ± 0.12^AB^	2953	5.89–6.27 pg/1.06-fold		–	1.48
Tupí Blanco/VAV6592	6.03 ± 0.50^AB^	2949	4.88–6.45 pg/1.32-fold	12.15–14.77 %	–	2.51
Pororó Grande/VAV6827	5.98 ± 0.09^AB^	2924	5.85–6.11 pg/1.04-fold		–	1.43
Azul/VAV6564	5.84 ± 0.43^BC^	2856	5.08–6.98 pg/1.37-fold	5.57–7.02 %	75	2.31*
Pororó Grande/VAV6562	5.79 ± 0.09^BCD^	2831	5.68–6.01 pg/1.06-fold		83	1.41*
Overo/VAV6559	5.79 ± 0.65^BCD^	2831	4.52–7.37 pg/1.63-fold	5.98–8.41 %	76	2.67*
Pipoca Colorado/VAV6567	5.74 ± 0.32^BCD^	2807	5.02–6.38 pg/1.27-fold	7.97–13.46 %	89	1.60*
Pipoca Colorado/VAV6607	5.69 ± 0.24^BCD^	2782	5.21–6.08 pg/1.17-fold	9.72–11.42 %	–	1.04
Colorado/VAV6573	5.68 ± 0.18^BCDE^	2777	5.23–5.97 pg/1.14-fold	7.27–9.76 %	64	1.85*
Blanco Ancho/VAV6560	5.64 ± 0.22^BCDE^	2758	5.01–6.10 pg/1.21-fold		77	2.38*
Rosado/VAV6565	5.58 ± 0.50^BCDEF^	2729	4.58–6.19 pg/1.35-fold	7.10–8.24 %	76	1.96*
Blanco Angosto/VAV6574	5.47 ± 0.13^CDEF^	2675	5.29–5.75 pg/1.08-fold		75	2.19*
Colorado/VAV6837	5.41 ± 0.32^DEF^	2645	4.93–5.86 pg/1.19-fold	6.76–9.07 %	–	2.52
Amarillo Ancho/VAV6569	5.37 ± 0.21^EF^	2626	5.01–5.80 pg/1.16-fold	6.00–9.19 %	80	2.38*
Pororó Chico/VAV6575	5.36 ± 0.24^EF^	2621	4.67–5.72 pg/1.22-fold	10.88–14.44 %	85	1.08
Amarillo Angosto/VAV6556	5.25 ± 0.16^F^	2567	4.91–5.62 pg/1.14-fold		66	2.17
Pipoca Amarillo/VAV6568	4.83 ± 0.72^G^	2362	3.96–5.92 pg/1.50-fold		85	1.18
Variegado/VAV6557	4.62 ± 0.66^G^	2259	3.81–5.81 pg/1.52-fold	5.30–8.63 %	74	2.37

Data of vegetative cycle (from sowing to anthesis of tassel flowers) were taken from Melchiorre *et al*. (2006). Data of seed mass (means weight of kernels) were estimated. Several data of seed mass were kindly donated by Ing. Agr. P. Melchiorre. The studied populations presented variations in their vegetative cycle (64–89 days) and seed mass (1.04–2.67 g) (Table 2).

**Figure 2.**
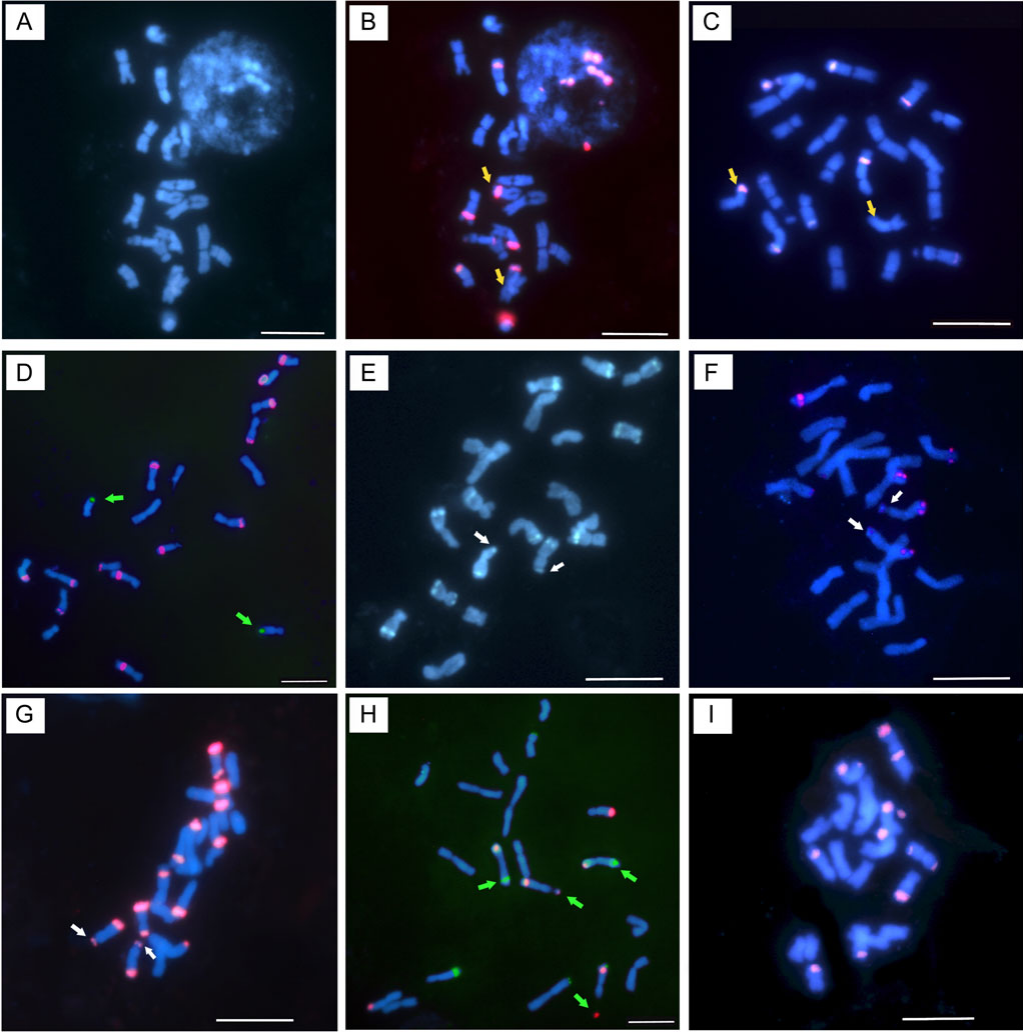
Fluorescent *in situ* hybridization on maize mitotic metaphases probed with 180-bp and TR-1. Rosado, VAV6565 (A and B); Overo, VAV6559 (C); Pipoca Colorado, VAV6607 (D); Pororó Chico, VAV6575 (E); Azul, VAV6564 (F); Tupí Amarillo, VAV6563 (G); Amarillo Angosto, VAV6569 (H); Variegado, VAV6557 (I). (A) DAPI staining. (B–I) Double exposure DAPI staining-FISH. Fluorescent *in situ* hybridization probes detected with Cy3 (red) and probes detected with anti-digoxigenin-FITC (green). (B and C) 180-bp (red). (D) TR-1 (green) and 180-bp (red). (E) TR-1 (green). (F) 180-bp (red). (G) 180-bp (red). (H) TR-1 (red) and 180-bp probe (green). (I) 180-bp (red). Green arrows indicate knobs composed only by TR-1 or 180-bp. White arrows show the satellites on the short arm of chromosome 6. Yellow arrows indicate heterozygosis for presence of knobs. Scale bars = 10 µm.

**Figure 3.**
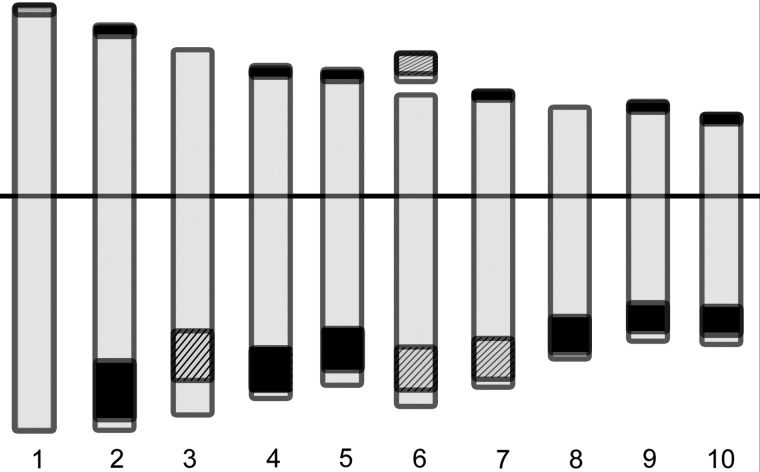
Representative idiogram of knob chromosome positions in maize. The hatched blocks indicate the knob chromosome positions that were always present. The black blocks depict the knob positions with inter- and intra-populational variations.

4′,6-Diamidino-2-phenylindole staining performed on mitotic metaphases showed variations in knob number (10–22), percentage of heterochromatin (5.30–16.10 %) and size and position of knobs (Fig. 2, Table 2). The population with the highest number of knobs was VAV6563 (14–22 knobs) on 11 different chromosome positions (Fig. 2, Table 3). Figure 2A and B shows a metaphase from VAV6565 with 10 conspicuous knobs and 1 little knob on the short arm of chromosome 1. Metaphases of VAV6575 and VAV6563 presented 16 conspicuous knobs with positive hybridization signal for both probes (Fig. 2D and G). Heterozygosis for the presence of knobs was detected (Fig. 2B and C). The studied populations presented knobs in 17 different chromosome positions (Table 3) that were schematized in a representative idiogram (Fig. 3).

**Table 3.** Ranges of numbers and chromosome positions of knobs. S, short arm; L, long arm; -sat, satellite; *detected in few individuals of each populations. ^1^Few individuals showed hybridization signals with only TR-1 sequence. ^2^Few individuals showed hybridization signals with only 180-bp sequence.

Maize landraces/vouchers	Range of numbers of knobs	Knob chromosome positions
Rosado/VAV6565	11–13	1S, 2S, 3L, 4S, 4L, 5L, 6L, 6-sat, 7L, 8L, 10L*
Variegado/VAV6557	11–13	1S, 3L, 4L, 5L, 6L, 6-sat, 7L, 8L
Overo/VAV6559	10–13	1S, 2L, 3L, 5L, 6L, 6-sat, 7L, 8L
Tupí Amarillo/VAV6563	14*–*22	1S, 2L, 3L, 4L, 4S, 6L, 6-sat, 7L, 7S, 8L, 9L
Pororó Chico/VAV6575	15–17	1S, 2L, 3L, 4L, 5L, 6L^1^, 6-sat, 7L, 7S, 9S, 10L*
Pipoca Colorado/VAV6607	15–19	1S, 2L, 3L, 4L, 4S*, 5S, 6L, 6-sat, 7L^1^, 9S, 10L*
Amarillo Ancho/VAV6569	10–12	1S, 2L, 3L, 4L^2^, 4S, 5L, 5S, 6L, 6-sat^1^, 7L^1^, 8L, 9L, 9S
Azul/VAV6564	10–13	1S, 3L, 4L, 4S*, 5S, 6L, 6-sat, 7L, 10L*
Colorado/VAV6837	11–13	1S, 2L*, 3L, 4L, 6L, 6-sat, 7L, 8L*

In FISH experiments, 180-bp and TR-1 knob sequences were probed on mitotic metaphases and both probes hybridized in very close juxtaposition in almost all knobs (Fig. 2, Table 3). Some individuals of VAV6607, VAV6575 and VAV6569 showed several knobs with hybridization signals with only TR-1 or 180-bp probes (Fig. 2D and H, Table 3).

**Figure 4.**
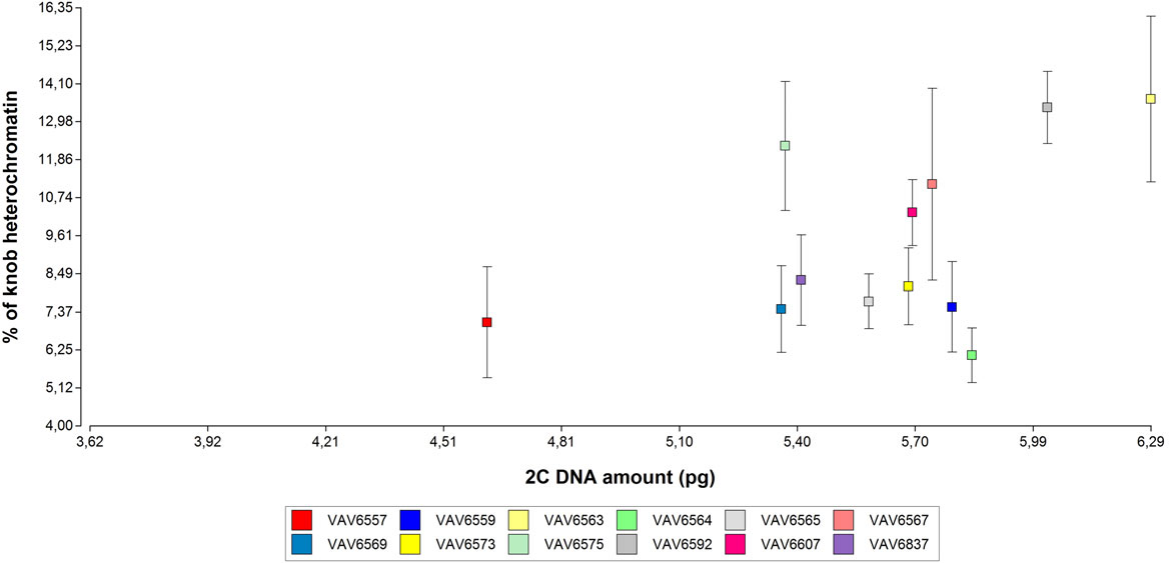
The relationship between the percentage of knob heterochromatin and the amount of 2C DNA (in pg), in each of the studied populations. The dots show the means values of the percentage of heterochromatin of each population. The whiskers represent the standard deviation.

A significant positive relationship between the number of knobs and the percentage of heterochromatin was found (*P* < 0.0001; Spearman coefficient = 0.88; df = 55). In Fig. 4 the percentage of heterochromatin and the 2C DNA amount were plotted; a significant correlation between both parameters was not found (*P* = 0.2380; Spearman coefficient = 0.35; df = 11).

**Figure 5.**
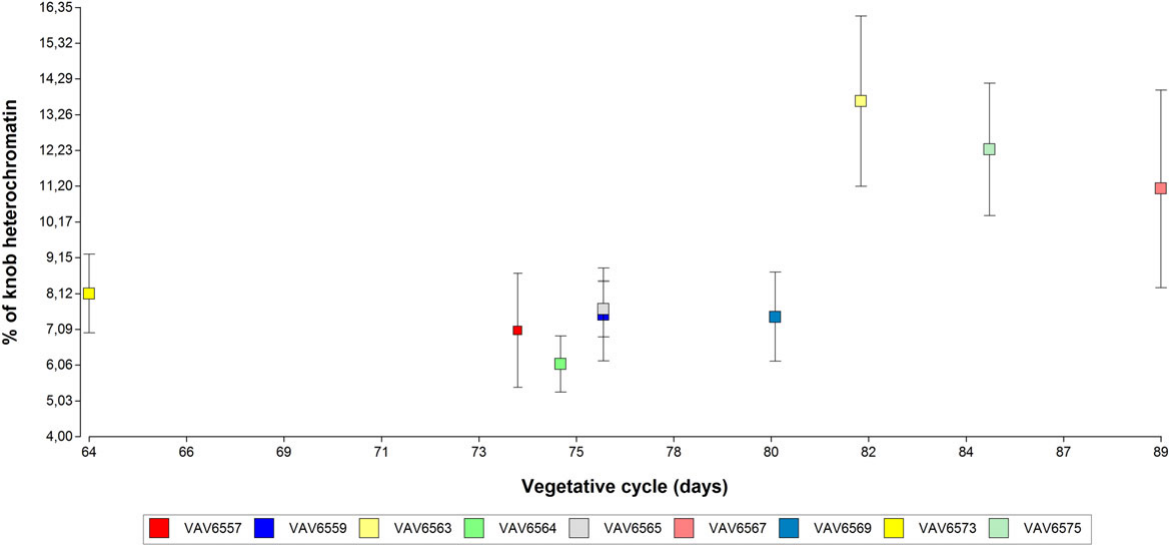
The relationship between the percentage of heterochromatin and the vegetative cycle length (in days). The dots show the means values of the percentage of heterochromatin of each population. The whiskers represent the standard deviation.

The length of the vegetative cycle showed a significant correlation with the percentage of heterochromatin (*P* = 0.0498; Spearman coefficient = 0.63 df = 7) (Fig. 5). The 2C DNA content did not show a significant correlation neither with the length of the vegetative cycle (*P* = 0.6409; Spearman coefficient = 0.13; df = 11) or with the seed mass (*P* = 0.4190; Spearman coefficient = 0.19; df = 18).

## Discussion

In this work, genome size variation (2C value) and cytological parameters (percentage of heterochromatin, number, positions and sequence composition of knobs) were studied in 20 populations of Guarani's maize landraces from NEA. Moreover, the relationship between these parameters and the vegetative cycle and seed mass were discussed. These relationships leave room for interpretations about the causes of genome size variation.

The 2C value varied from 4.62 to 6.29 pg, representing 36.15 % of inter-populational variation (1.98-fold). A similar variation was found in populations with a wide range in altitude of cultivation by Rosato *et al*. (1998) in northwestern Argentinean landraces and by Díez *et al*. (2013) in Mexican landraces. The variation in genome size was also reported in populations from the USA and Mexico (Laurie and Bennett 1985; Rayburn *et al*. 1985; Porter and Rayburn 1990; Rayburn and Auger 1990*a*, *b*). In the present study, significant intra-populational genome size variations were also found, being 1.63-fold and 1.08-fold, the maximum and the minimum, variation detected among populations. In a recent review of Greilhuber and Leitch (2013), the existence of intra-specific genome size variation was discussed. These authors pointed out that flow cytometry is the appropriate method for detecting intra-specific genome size variation. Doležel *et al*. (1998) found a linear significant relationship between the data measured by flow cytometry and that acquired by Feulgen densitometry.

Previous studies reported that, in *Zea*, the differences in the DNA content were related with variations in the number and size of knobs/heterochromatic bands (Laurie and Bennett 1985; Rayburn *et al*. 1985; Tito *et al*. 1991, Poggio *et al*. 1998). In the present work, DAPI staining and FISH allowed us to detect inter- and intra-populational variations in the number, size and sequence composition of the knobs. The inter-populational variation in the number of knobs (10–22) and the percentage of heterochromatin (5.3–16.1 %) were found, and a significant relationship between these parameters was also detected. The population VAV6563 with the maximum number of knobs and the greater percentage of heterochromatin possesses the highest genome size (2C = 6.29 pg). Besides, the population VAV6557 with a low number of knobs and low percentage of heterochromatin possesses the lowest genome size (2C = 4.62 pg). However, two populations (VAV6559 and VAV6564), with a low number of knobs and low percentage of heterochromatin, presented intermediate 2C values (5.7–5.9 pg). While genome size increases in populations with a high percentage of heterochromatin, a significant correlation between these parameters was not found. This indicates that satellite DNA, which conforms the heterochromatin and contributes to the genome size of maize, is not the only source of variation. The amplifications or deletions of dispersed sequences of genome, such as retroelements and other repetitive sequences, which in maize makes up over 70 % of the nuclear genome, are also relevant (SanMiguel and Bennetzen 1998; Meyers *et al*. 2001).

The intra-populational variation in the number of knobs and the percentage of heterochromatin was also detected. Major intra-populational variation was observed in VAV6563 (14*–*22 knobs; 10.33–16.10 % of heterochromatin). This variation between individuals of the same population could be explained by the heterozygosity for the presence/absence observed in each knob chromosome position, although the variation in other repetitive DNA sequences could not be discarded.

Another source of intra-specific variation was the chromosome position and sequence composition of the knobs. 4′,6-Diamidino-2-phenylindole staining allowed us to detect that the knobs are located not at random, in 17 different chromosomal positions, which varied among the populations. Moreover, the FISH experiments demonstrated that while, in most of the analysed populations, the knobs comprise 180-bp and TR-1 sequences in very close juxtaposition, several populations presented knobs with only one knob sequence. Therefore, the knob positions and their sequence composition are useful tools for the cytological characterization of the landraces here studied.

Many works focused on the relationships between genome size and phenotype at whole plant levels (Bennett and Leitch 2005; Greilhuber and Leitch 2013). Genome size and vegetative cycle or flowering time were related in several groups of plants (revisited in Greilhuber and Leitch 2013). In the populations analysed here, a significant correlation between the DNA content and vegetative cycle was not found. Interestingly, the increase in the length of the vegetative cycle was positively related with the increase in the percentage of heterochromatin. These results could be explained if the knob heterochromatin is the last component to complete DNA replication as it was postulated (Pryor *et al*. 1980; Greilhuber and Leitch 2013). These authors consider that the increased packaging of heterochromatin leads to a longer DNA synthesis phase and hence longer cell cycle time. Several authors determined that, in maize, the percentage of heterochromatin is positively correlated with the vegetative period and considered that the decrease in heterochromatic knobs could be an adaptation to a shorter growing season and the result of the artificial selection by man (Rayburn *et al*. 1985; Price 1988; Tito *et al*. 1991). The populations analysed in this work grow in a small and restricted eco-geographical area, without significant differences in altitude, climatic or biological conditions, but they are isolated by temporal reproductive barriers due to differences in their flowering time. This isolation would be maintained by the artificial selection by man, because the farmers select against corncobs with an intermediate morphology among landraces. In fact, when the artificial selection against hybrids between two varieties of maize was performed, the level of spontaneous inter-varietal hybridization decreases and the reproductive isolation was enhanced, mainly due to greater differences between varieties in their flowering time (Grant 1981).

Since the vegetative cycle time would be optimized via selection for an appropriate percentage of heterochromatin, the positive correlations found between them allows to propose an adaptive effect to heterochromatin.

Another phenotypic characteristic that has been related to genome size is the seed mass (Bennett 1987; Bennett and Leitch 2005; Knight *et al*. 2005; Beaulieu *et al*. 2007). Positive relationships between the DNA amount and seed weight have been reported in a large number of species (Bennett 1972, 1987; Caceres *et al*. 1998; Knight and Ackerly 2002; Bennett and Leitch 2005). In the landraces of maize here studied, a relationship between the DNA content and seed weight was not detected, because numerous other factors could influence the seed weight independently of genome size. In fact, Knight *et al*. (2005) point out that not in all cases this relationship could be linear.

Multivariate analysis including morphological, phenological and reproductive traits (Melchiorre *et al*. 2006) and nuclear microsatellite characterization (Bracco *et al*. 2009, 2012) were performed in the NEA landraces here studied. Both studies allowed us to distinguish two groups of landraces, the popcorns and the flourys. In the present work, popcorn populations showed higher percentage of heterochromatin, whereas the floury populations had the lowest. Therefore, the percentage of heterochromatin could be another discriminating character between popcorn and floury landraces.

## Conclusions

The maize Guarani’s landraces from NEA have inter-population and even intra-population variations in their genome size, as well in the percentage of heterochromatin, the number, and position of chromosomes and sequence compositions of the knobs. The variation found in 2C values could be due to the heterozygosity for the presence/absence observed in each knob chromosome position and percentage of heterochromatin variation, however the variation in other repetitive DNA sequences could not be discarded.

The positive correlations between the length of the vegetative cycle and percentage of heterochromatin, found in the present work, allowed attributing an adaptive effect to heterochromatin since vegetative cycle time would be optimized via selection for an appropriate percentage of heterochromatin.

## Sources of Funding

The funding was provided by the grants from the Consejo Nacional de Investigaciones Científicas y Técnicas (CONICET-PIP 00342), Universidad de Buenos Aires (UBACYT) and the Agencia Nacional de Producción Científica y Tecnológica-SECyT (PICT 1665).

## Contributions by the Authors

All authors contributed to the experimental design, data analysis and manuscript preparation.

## Conflict of Interest Statement

None declared.
